# Voting based double-weighted deterministic extreme learning machine model and its application

**DOI:** 10.3389/fnbot.2023.1322645

**Published:** 2023-11-21

**Authors:** Rongbo Lu, Liang Luo, Bolin Liao

**Affiliations:** ^1^College of Computer and Artificial Intelligence, Huaihua University, Huaihua, China; ^2^College of Computer Science and Engineering, Jishou University, Jishou, China

**Keywords:** intelligent learning model, neural network, machine recognition classification, weights determination, machine-assisted diagnosis

## Abstract

This study introduces an intelligent learning model for classification tasks, termed the voting-based Double Pseudo-inverse Extreme Learning Machine (V-DPELM) model. Because the traditional method is affected by the weight of input layer and the bias of hidden layer, the number of hidden layer neurons is too large and the model performance is unstable. The V-DPELM model proposed in this paper can greatly alleviate the limitations of traditional models because of its direct determination of weight structure and voting mechanism strategy. Through extensive simulations on various real-world classification datasets, we observe a marked improvement in classification accuracy when comparing the V-DPELM algorithm to traditional V-ELM methods. Notably, when used for machine recognition classification of breast tumors, the V-DPELM method demonstrates superior classification accuracy, positioning it as a valuable tool in machine-assisted breast tumor diagnosis models.

## 1 Introduction

Extreme Learning Machine (ELM) (Huang et al., 2004) is a powerful machine learning algorithm that has emerged as a popular alternative to traditional neural networks [such as Back-Propagation (Haykin, 1998) algorithm (BP) and Levenberg Marquardt (Levenberg, 1944; Marquardt, 1963) algorithm] due to its speed, simplicity, and high performance. ELM is a single-layer feedforward neural network that uses random weight initialization and least-squares optimization to learn from input data (Huang et al., 2006). The algorithm has shown remarkable results in a wide range of applications, from image recognition (Tang et al., 2015) and speech processing (Han et al., 2014) to financial forecasting (Fernández et al., 2019) and anomaly detection (Huang et al., 2015).

One drawback of the ELM algorithm is that the learning parameters of the hidden nodes are randomly assigned and remain unchanged during training, which may lead to a significant impact on its predictive performance and algorithm stability (Gao and Jiang, 2012; Lu et al., 2014). ELM might misclassify certain samples, particularly those near the classification boundaries. In an attempt to address this issue, Cao et al. (2012) proposed a voting-based variant of ELM, referred to as V-ELM. The main idea behind V-ELM is to perform multiple independent ELM trainings instead of a single training, and then make the final decision based on majority voting. However, this approach does not fundamentally resolve the problem of random determination of ELM's various parameters.

Zhang et al. (2014) have highlighted that the performance of Extreme Learning Machine (ELM) is not always optimal when the input weights and hidden layer biases are chosen entirely at random. This randomness is also a significant factor contributing to the redundancy of neurons in the hidden layer of the ELM algorithm (Zhu et al., 2005). In response, scholars have proposed the use of swarm intelligence optimization (Lahoz et al., 2013; Figueiredo and Ludermir, 2014; Zhang et al., 2016), pruning methods (Miche et al., 2009, 2011), and adaptive algorithms (Pratama et al., 2016; Zhao et al., 2017) to optimize the ELM algorithm and enhance its overall performance. However, in practical applications, although these algorithms do succeed in optimizing the number of hidden layer neurons, they introduce a plethora of hyperparameters that typically require iterative optimization, thereby increasing the computational complexity of the algorithm and rendering it challenging to address real-time problems with high time constraints. To tackle this issue, this paper presents an improved algorithm known as Voting based double Pseudo-inverse weights determination Extreme Learning Machine (V-DPELM). The core concept of V-DPELM lies in the stochastic determination of output weights, while input weights are obtained through pseudoinverse calculations. Subsequently, the pseudo-inverse method is employed again to determine optimal output weights, ensuring that both input and output weights are optimal. The obtained DPELM algorithm is subjected to multiple independent trainings, and the final decision is made based on majority voting.

In the 21st century, breast cancer is increasingly recognized as a significant factor negatively impacting the overall quality of life for women worldwide. According to statistics from the World Health Organization (WHO), approximately 1.5 million women suffer greatly from the torment of breast cancer, with approximately 500,000 losing their lives to this disease (Fahad Ullah, 2019). The incidence and mortality rates of breast cancer exhibit a clear and alarming upward trend each year. Research has demonstrated the paramount importance of timely detection, diagnosis, and initiation of treatment in achieving favorable therapeutic outcomes for breast cancer (Lee et al., 2019; Aldhaeebi et al., 2020). Ten crucial features, including symmetry and fractal dimension of breast tumor lesions, play a vital role in determining the nature of the tumor, whether benign or malignant (Wang et al., 2016, 2019). Therefore, it is possible to extract relevant features closely associated with tumor characteristics from acquired patient samples. By employing the proposed V-DPELM algorithm for parameter optimization and subsequent breast tumor classification, the obtained classification and identification results can provide valuable references, assisting physicians in making diagnostic decisions and offering more accurate and rational assessments of patients' conditions.

## 2 V-DPELM algorithm design

In the section, we first review the basic concept of the traditional ELM algorithm in Section 2.1. Then, we analyzed the DPELM algorithm in Section 2.2. Finally, the new proposed V-DPELM algorithm will be presented in Section 2.3.

### 2.1 Brief review of ELM

**Figure 1:**
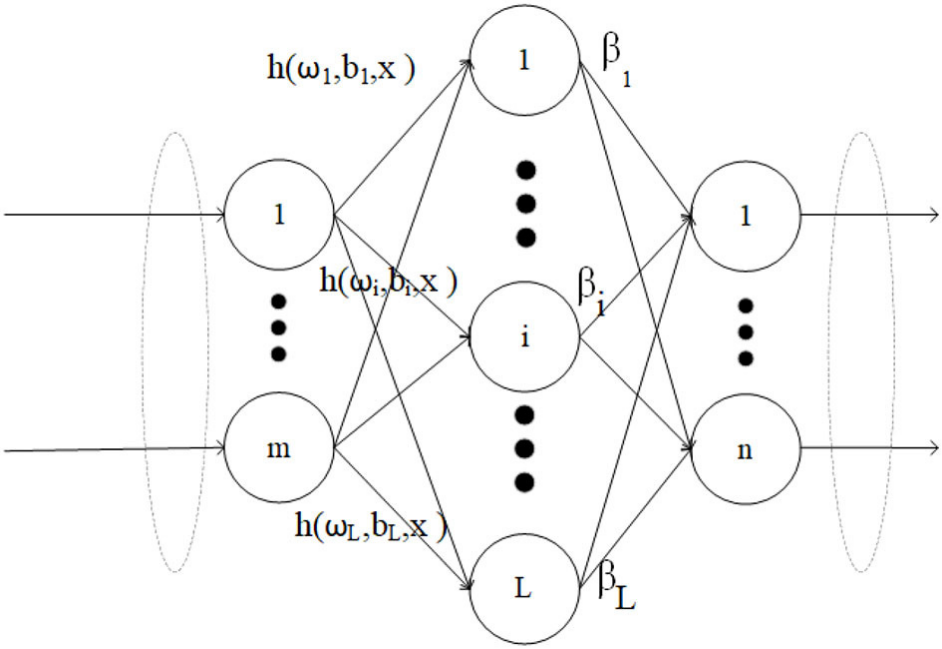
ELM network structure.

Extreme Learning Machine (ELM) is suitable for generalized Single Hidden Layer Feedforward Networks (SLFN). The structure of traditional ELM is similar to SLFN, consisting of three layers: input layer, hidden layer, and output layer. The essence of ELM is that it does not require tuning the hidden layer of SLFN. The structure of ELM is shown in Figure 1.

In the context of N arbitrary training samples ${\left\{\left(x_{i},t_{i}\right)\right\}}_{i=1}^{N}$, where each sample ${\text{x}}_{i}={\left(x_{i1},x_{i2},...,x_{in}\right)}^{\text{T}}\in {\mathbb{R}}^{n}$, ${\text{t}}_{i}={\left(t_{i1},t_{i2},...,t_{im}\right)}^{\text{T}}\in {\mathbb{R}}^{m}$, the resulting output of the ELM with L hidden nodes can be expressed as follows:


(1) $$\begin{array}{l} {\text{t}}_{i}=\overset{L}{\underset{j=1}{\sum }}\beta_{j}h\left(\omega_{j},b_{j},{\text{x}}_{i}\right),i=1,2,...,N \end{array}$$

Here, ω_*j*_ = (ω_*j*1_, ω_*j*2_, ..., ω_*jn*_) represents the weight vector of the *j*th neuron in the input layer, and *b*_*j*_ is the bias associated with the *j*th neuron. *h*(.) indicates the activation function. Furthermore, β_*j*_ denotes the linked weights between the *j*th hidden neurons and output neurons, β_*j*_ = (β_*j*1_, β_*j*2_, ..., β_*jm*_).

For all *N* samples, the equivalent canonical form of linear equation (1) can be expressed as:


(2) $$\begin{array}{l} \text{H}\beta =\text{T}, \end{array}$$

In Equation (2), T represents the desired output matrix for the training samples, and


$$\begin{array}{l} \text{H}=\left[\begin{array}{ccc} h\left(\omega_{1},b_{1},{\text{x}}_{1}\right) & \ldots & h\left(\omega_{L},b_{L},{\text{x}}_{1}\right)\\ \vdots & \ddots & \vdots \\ h\left(\omega_{1},b_{1},{\text{x}}_{N}\right) & \ldots & h\left(\omega_{L},b_{L},{\text{x}}_{N}\right) \end{array}\right] \end{array}$$

is the randomized matrix mapping. It is worth noting that the parameters (ω_*j*_, *b*_*j*_) of the hidden layer neurons are randomly generated and remain fixed throughout the entire training process of ELM.

The ELM algorithm can be summarized as three steps as follow.

- Step 1: Randomly generate parameters for the hidden layer nodes.
- Step 2: Calculate the output matrix *H* of the hidden layer.
- Step 3: Calculate the output weight using $\tilde{\beta }={\text{H}}^{\text{†}}\text{T}$, † represents the pseudo-inverse of the matrix.

### 2.2 DPELM learning algorithm

Due to the random determination of input weights in traditional ELM, it has resulted in low classification accuracy and an issue of too many hidden layer nodes. Therefore, this section introduces a new method for determining ELM's weights, referred to as the double pseudo-inverse weights determination ELM (DPELM), aiming to enhance its classification accuracy and achieve a more stable structure. DPELM is similar to the traditional ELM network structure, which consists of input layer, hidden layer and output layer. Upon a more comprehensive analysis of the traditional ELM principle, Equation 1 can be reformulated as follows:


(3) T=βh(ΩX−B),

where $\text{T}=\left[{\text{t}}_{1},{\text{t}}_{2},...,{\text{t}}_{N}\right]\in {\mathbb{R}}^{m\times N}$, $\text{X}=\left[{\text{x}}_{1},{\text{x}}_{2},...,{\text{x}}_{N}\right]\in {\mathbb{R}}^{n\times N}$, $\text{B}=\left[{\text{b}}_{1},{\text{b}}_{2},...,{\text{b}}_{N}\right]\in {\mathbb{R}}^{L\times N}$, **β** and **Ω** represent the output weight matrix and the input weight matrix, respectively. Where


$$\begin{array}{l} \begin{array}{cc} \beta & =\left[\begin{array}{cccc} \beta_{11} & \beta_{12} & \ldots & \beta_{1L}\\ \beta_{21} & \beta_{22} & \ldots & \beta_{2L}\\ \vdots & \vdots & \ddots & \vdots \\ \beta_{m1} & \beta_{m2} & \ldots & \beta_{mL} \end{array}\right]\in {\mathbb{R}}^{m\times L},\\ \Omega & =\left[\begin{array}{cccc} \omega_{11} & \omega_{12} & \ldots & \omega_{1n}\\ \omega_{21} & \omega_{22} & \ldots & \omega_{2n}\\ \vdots & \vdots & \ddots & \vdots \\ \omega_{L1} & \omega_{L2} & \ldots & \omega_{Ln} \end{array}\right]\in {\mathbb{R}}^{L\times n}. \end{array} \end{array}$$

**Derivation process:** Assuming the bias *B* and output weight β are randomly generated within the interval [a1, a2], and the activation function *h*(·) is strictly monotonous, the ideal **Ω** should be equal to **Ω** = (*h*^−1^(**β**^†^**T**)+**B**)**X**^†^.

Since *B* and β are randomly generated, multiplying both sides of Equation 3 by **β**^†^ results in:


(4) $$\begin{array}{l} \beta^{\text{†}}\text{T}=\beta^{\text{†}}\beta h\left(\Omega \text{X}-\text{B}\right)=h\left(\Omega \text{X}-\text{B}\right). \end{array}$$

By finding the inverse function of the activation function *h*(·), we can obtain:


$$h^{-1}(\beta^{†}T)=\Omega X-B,$$

The above equation can be rewritten as:


(5) $$\Omega X=h^{-1}(\beta^{†}T)+B.$$

Finally, multiplying equation 5 by **X**^†^ simultaneously results in


$$\Omega XX^{†}=(h^{-1}(\Lambda^{†}Y)+\Phi )X^{†},$$

namely,


$$\Omega =\left(h^{-1}\left(\beta^{\text{†}}\text{T}\right)+\text{B}\right){\text{X}}^{\text{†}}.$$

This concludes the proof.

Once the optimal **Ω** has been determined, the formula $\tilde{\beta }=T{\left(h(\Omega X-B)\right)}^{†}$ can be employed to compute the value of $\tilde{\beta }$.

### 2.3 V-DPELM model training process

Based on theoretical principles, the specific training process for V-DPELM model is outlined as follows:

- Step 1: Given a sample dataset $ℵ={\left\{\left({\text{x}}_{i},{\text{t}}_{i}\right)|{\text{x}}_{i}\in {\mathbb{R}}^{n},{\text{t}}_{i}\in {\mathbb{R}}^{m}\right\}}_{i=1}^{N}$, where **x**_*i*_, **t**_*i*_, *N* represent the input vector, target vector, and the total number of samples, respectively. This step introduces essential parameters, including the hidden node output function *h*(ω, *b, x*), the count of hidden nodes *L*, and the number of independent training repetitions *K*.
- Step 2: Randomly initialize output weights β and hidden layer biases *B* within the interval [a1, a2].
- Step 3: In the case where the training sample is determined, the optimal input weights Ω are computed using the formula **Ω** = (*h*^−1^(**β**^†^**T**)+**B**)**X**^†^.
- Step 4: Subsequently, upon obtaining the optimal input weights Ω, the optimal output weights $\tilde{\beta }$ are determined as $\tilde{\beta }=T{\left(h(\Omega X-B)\right)}^{†}$.
- Step 5: Repeat steps 2 to 4 for a total of *K* times to get *K* independent DPELMs model. Then, perform test tasks on these DPELMs, and the final result is obtained by aggregating the test results using a voting strategy.

**Figure 2:**
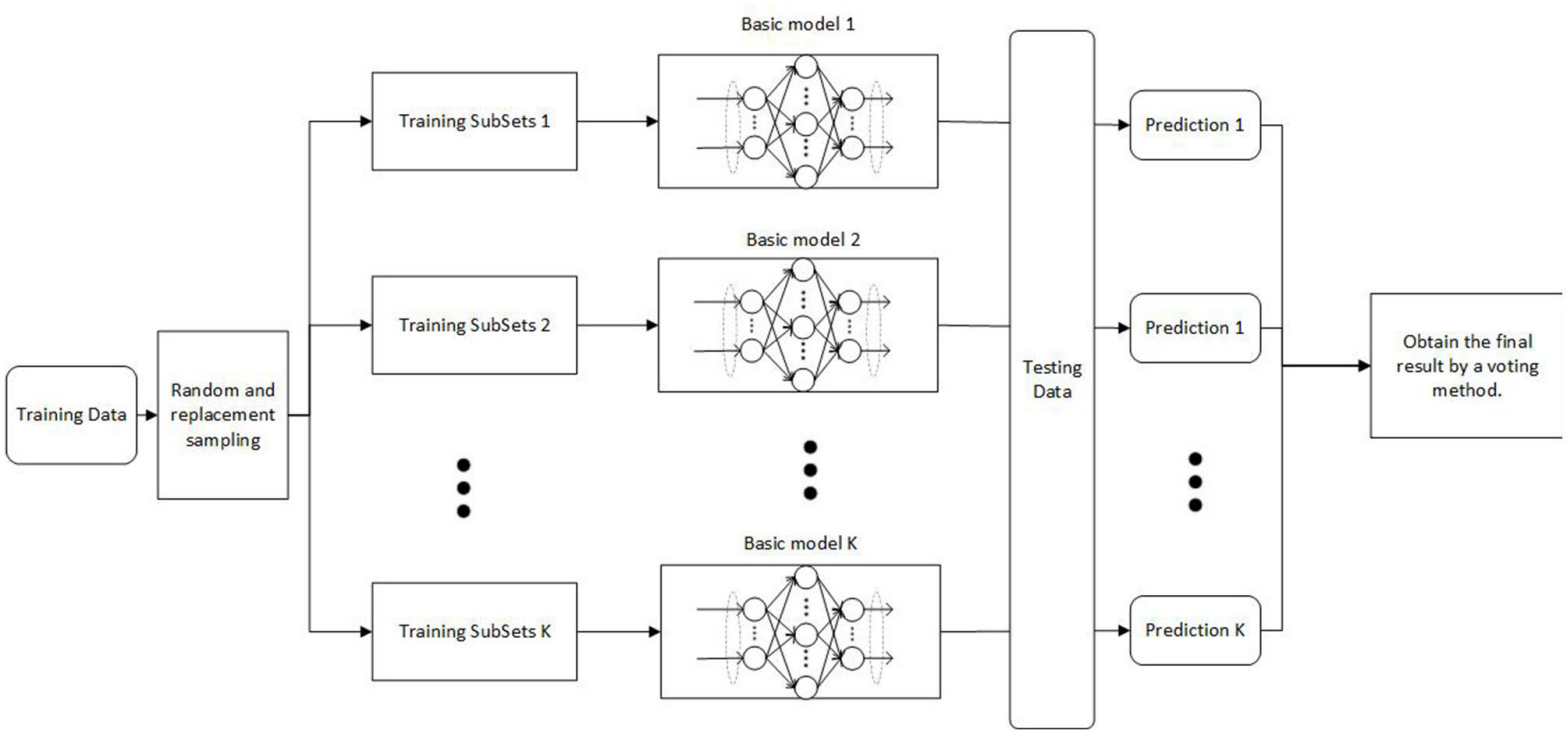
V-DPELM network structure.

The network structure of V-DPELM model is shown in Figure 2. Algorithm 1 provides a specific introduction to the pseudo code of the V-DPELM method.

**Algorithm 1:** V-DPELM.

**Input:** $ℵ={\left\{\left({\text{x}}_{i},{\text{t}}_{i}\right)|{\text{x}}_{i}\in {\mathbb{R}}^{n},{\text{t}}_{i}\in {\mathbb{R}}^{m}\right\}}_{i=1}^{N}$, hidden active function *h*(ω, *b, x*), hidden nodes *L*, independent training repetitions *K*, zero valued vector $S_{K}\in {\mathbb{R}}^{m}$;
**Output:** *TestingAccuracy*;
1: *Set* *k* = 1;
2: **while** *k* ≤ *K* **do**
3: Randomly assign the learning parameters $\left(\beta_{i}^{k},b_{i}^{k}\right)$ of the *k*th DPELM;
4: Calculate the input weight ω^*k*^;
5: Calculate the hidden layer output matrix *H*^*k*^;
6: Calculate the output weight $\tilde{\beta^{k}}$, $\tilde{\beta }=T{\left(h(\Omega X-B)\right)}^{†}$;
7: *k* = *k*+1;
8: **end while**
9: *c* = *a*+*b*;
10: **for all** testing sample*x*^*test*^ **do**
11: *Set* *k* = 1;
12: **while** *k* ≤ *K* **do**
13: using the *k*th trained basic DPELM with leaning parameters $\left(\beta_{i}^{k},b_{i}^{k},\omega_{i}^{k}\right)$ to predict the label of the testing sample *x*^*test*^;
14: Each generated prediction result is then stored in *S*_*K*_;
15: *k* = *k*+1;
16: **end while**
17: The final class label of testing sample *x*^*test*^ is $c^{\text{test}}=\arg\underset{j\in \left[1,\cdots \;,m\right]}{\max}\left\{S_{K,x^{\text{test}}}\left(j\right)\right\}$
18: **end for**

## 3 Experimental results and analysis

This section randomly selects 12 datasets from the UCI database to assess the classification performance of the improved Extreme Learning Machine algorithm. All experiments in this paper were conducted using Matlab 2016(a) on a regular PC with an Intel(R) Core(TM) i5-12500H CPU running at 3.60GHz and 16GB of memory.

### 3.1 Experimental description

The present text conducts a series of experiments to evaluate the performance of the algorithm from various perspectives, including the efficacy of its categorization, the precision of its predictions, the requisite count of neurons within its hidden layers, and the stability of its resultant outputs. The datasets utilized in this research were sourced from the UCI (University of California, Irvine) repository, encompassing both binary classification and multi-classification datasets. It is important to note that the training and test data within each dataset were randomly shuffled for each simulation experiment, ensuring unbiased evaluations. Detailed specifications of these 12 datasets are presented in Table 1.

**Table 1:** Specifications of classification datasets.

**Datasets**	**Attributes**	**Classes**	**Samples**	**Testing data**
SL	35	19	215	92
Iris	4	3	100	50
Wine	13	3	100	78
Liver disorders (LD)	6	2	240	105
Pima Indians diabetes (PID)	8	2	537	231
Innosphere	34	2	220	95
Diabetes	8	2	576	191
Balance	4	3	400	225
Ecoli	7	8	100	236
Waveform	21	3	3000	2000
Live	6	2	200	145

### 3.2 Experimental results and analytical discussion

**Figure 3:**
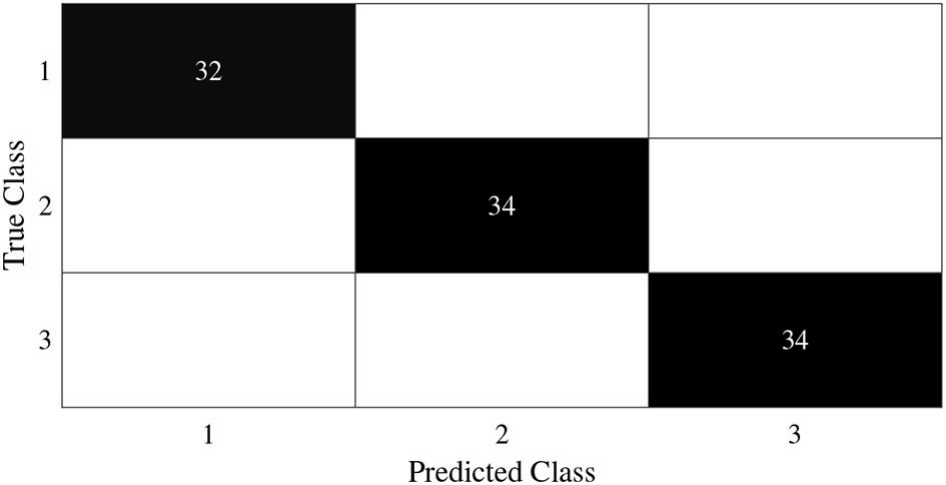
Training confusion matrix of Iris dataset.

**Figure 4:**
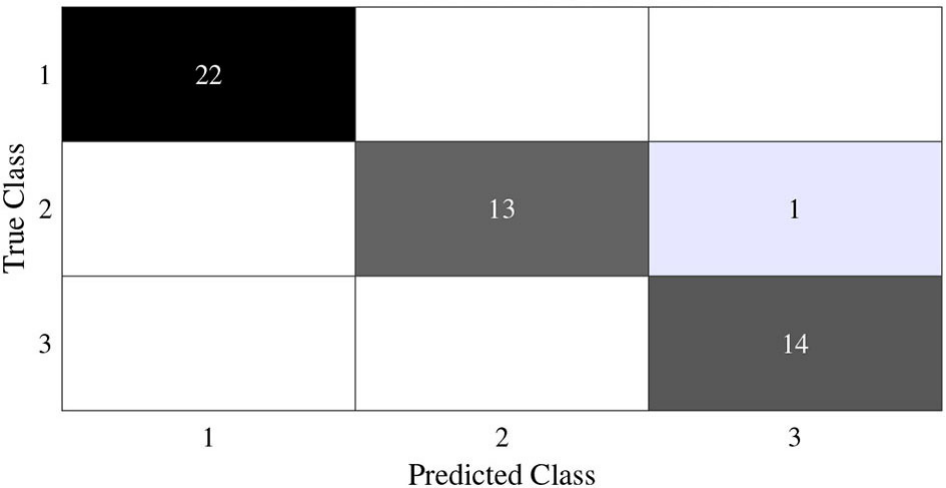
Test confusion matrix of Iris dataset.

**Figure 5:**
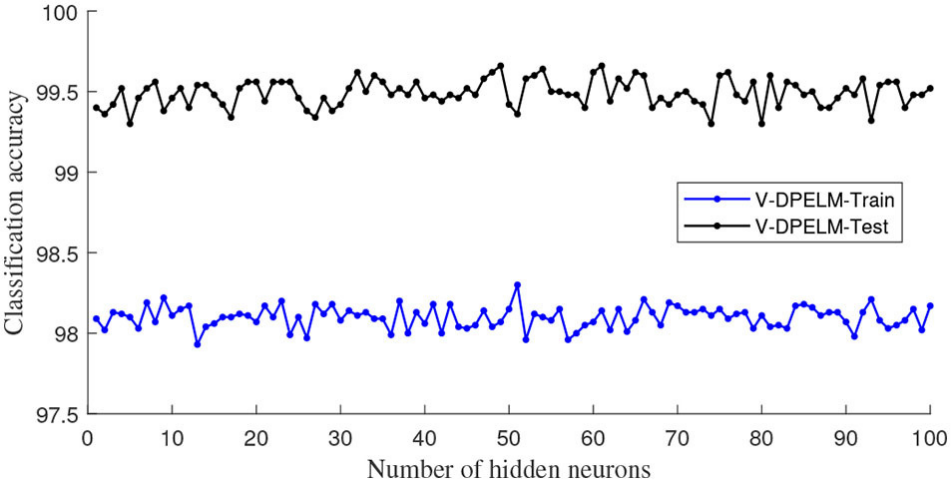
V-DPELM classification accuracy for Iris dataset.

In this subsection, we begin by employing the Iris dataset, the features of which are displayed in Table 1, to ascertain the efficacy of the V-DPELM algorithm. The corresponding outcomes are illustrated through Figures 3–5 and Table 2. Figures 3, 4 depict the graphs of the confusion matrix. Within these figures, the values along the diagonal of the matrix signify the correctly classified samples, whereas those located elsewhere indicate the misclassified samples.

**Table 2:** Classification performance of V-DPELM with different hidden layer neuron numbers in the Iris Dataset.

**V-DPELM**	**Accuracy rate (** ***%*** **)**		**Neurons**
	**Training**	**Testing**	
	98.09	99.40	1
	98.02	99.36	2
	98.13	99.42	3
	98.12	99.52	4
	98.10	99.30	5
	98.11	99.46	10
	98.07	99.56	20
	98.15	99.42	50
	98.17	99.52	100

It is evident that V-DPELM exhibits noteworthy proficiency in performing classification tasks, both in testing and training scenarios. Furthermore, as evident from Figure 5, the optimal classification accuracy reaches approximately 99.5% during testing and 98% during training. Notably, Figure 5 unveils a significant observation: the generalization performance of V-DPELM remains stable even with a modest number of hidden-layer neurons. This finding is corroborated by Table 2. Specifically, when the count of hidden-layer neurons is set to 3, optimal and consistent classification accuracy is achieved. This phenomenon holds true for other cases as well.

Regarding Table 2, there is an additional aspect that requires elucidation. In the context of assessing the presented growth methodology, the number of hidden-layer neurons in V-DPELM is tuned either manually, with an increment of 1, or automatically through the growth method. As demonstrated in the table, the proposed growth method effectively identifies the optimal structure for V-DPELM. Consequently, the effectiveness of V-DPELM in pattern classification is preliminarily affirmed.

The impact of the number of neurons in the hidden layer on the predictive performance of both the traditional V-ELM and the algorithm proposed in this study is investigated through experimental comparisons. Initially, a subset of samples from each dataset is selected as training and testing data, with the division between them fixed throughout the experiment. The growing method is employed to determine the number of neurons in the hidden layer, where the accuracy is observed after each addition of one neuron. The corresponding algorithm is considered to have the best network structure when the accuracy remains unchanged or the change falls below a predefined threshold. Subsequently, the ELM algorithm and the algorithm proposed in this paper are executed 100 times within the optimized network structure, and the average classification accuracy is computed using the test dataset. In this experiment, the tangent function (tan) is chosen as the activation function, with its inverse function being the arctangent function (arctan). The comparative analysis of classification accuracy for different algorithms and the required number of neurons in the hidden layer to achieve the highest classification accuracy are presented in Table 3.

**Table 3:** Comparisons of classification accuracy and number of hidden layer neurons of different algorithms.

**Datasets**	**Testing (** ***%*** **)**		**Hidden layer neurons**	
	**V-ELM**	**V-DPELM**	**V-ELM**	**V-DPELM**
SL	90.25	**92.30**	83	63
Iris	98.42	**99.56**	15	9
Wine	99.38	**99.93**	30	10
Liver Disorders (LD)	73.24	**73.33**	24	7
Pima Indians Diabetes (PID)	81.07	**83.37**	35	30
Innosphere	91.35	**92.88**	47	5
Diabetes	70.96	**81.23**	40	5
Zoo	96.61	**98.22**	20	10
Balance	90.49	**92.08**	40	30
Ecoli	85.23	**89.15**	20	10
Waveform	76.37	**78.31**	80	30
Liver	71.56	**73.79**	20	10

Bold values indicate the maximum value.

From Table 3, it can be observed that the algorithm proposed in this paper outperforms the traditional V-ELM algorithm in terms of classification performance, both in binary datasets and multi-classification datasets. The proposed algorithm achieves higher classification accuracy with fewer neurons in the hidden layer, resulting in a simpler network structure. This indicates that the analytical weight initialization method employed in this paper yields superior results compared to the random weight initialization method. Furthermore, to further analyze the impact of algorithm parameters on classification performance and algorithm stability, this study selects one dataset each from binary and multi-class problems for performance comparison.

**Figure 6:**
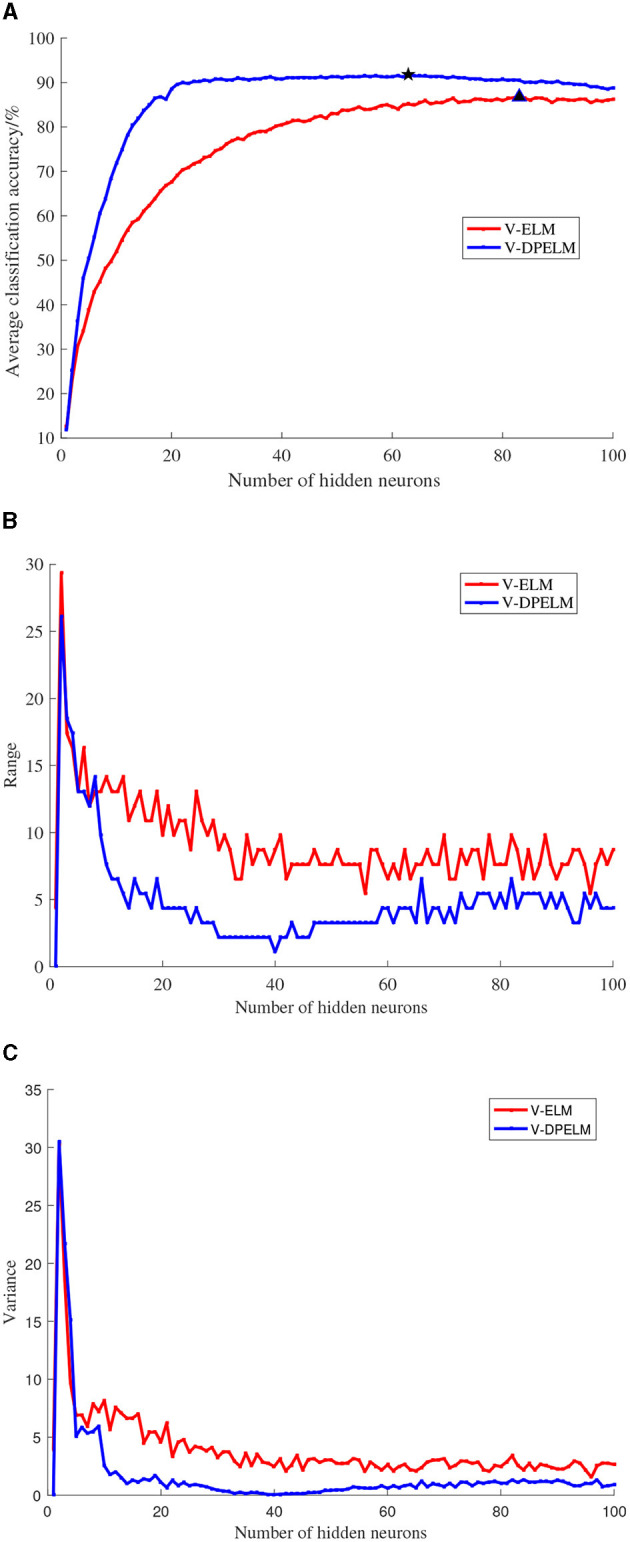
SL data set comparison experiment results. **(A)** Changes in classification accuracy. **(B)** Changes in range. **(C)** Changes in variance.

**Figure 7:**
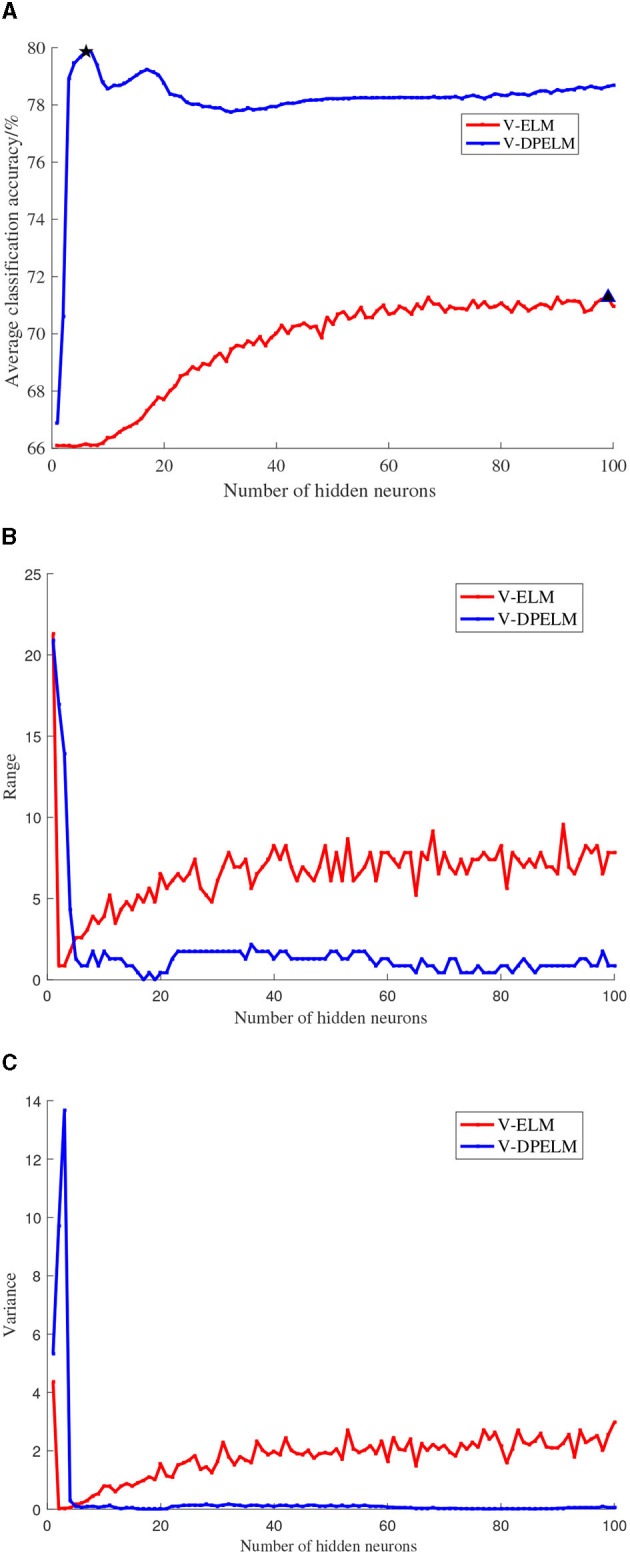
Diabetes data set comparison experiment results. **(A)** Changes in classification accuracy. **(B)** Changes in range. **(C)** Changes in variance.

The SL dataset, a multi-class dataset, and the Diabetes dataset, a binary classification dataset, are selected for this study. The training and testing sets for both datasets are fixed and unchanged throughout the experiments. The number of neurons in the hidden layer is set to increment from 1 to 100. For each additional neuron, the ELM algorithm and the algorithm proposed in this paper are executed 100 times. The experimental results are analyzed in terms of the mean, variance, and range, as depicted in Figures 6, 7. In these figures, the positions indicated by black pentagons and triangles represent the locations where each algorithm achieves the highest classification accuracy.

Observing Figures 6A, 7A, it becomes evident that the increase in the number of neurons in the hidden layer leads to an initial rapid rise in prediction accuracy for both the traditional V-ELM algorithm and the algorithm proposed in this paper. However, after reaching a certain point, the accuracy levels off or slightly declines. By considering the experimental findings and the Theorem presented in Huang et al. (2006), it can be deduced that the algorithm proposed in this study shares similar characteristics with the traditional V-ELM algorithm. Specifically, as the number of neurons in the hidden layer increases, the algorithm's fitting performance improves. Nevertheless, beyond a critical threshold, further augmenting the number of hidden neurons may cause overfitting on the training samples, resulting in a slower or even decreasing classification accuracy on the test samples.

Furthermore, a thorough examination of Figures 6, 7 reveals that, in both the multi-class SL dataset and the binary Diabetes dataset, the proposed algorithm demonstrates a faster rate of average classification accuracy improvement compared to the conventional V-ELM algorithm. Remarkably, achieving this progress requires a smaller number of neurons in the hidden layer. Additionally, the analysis of variance and range reveals that the proposed algorithm exhibits lower values for both metrics compared to the traditional V-ELM algorithm on the SL and Diabetes datasets. This finding suggests that the proposed algorithm possesses superior stability in comparison to the traditional V-ELM algorithm.

## 4 Application of V-DPELM in the diagnosis of breast tumors

In order to further validate the accuracy of voting based double pseudo-inverse weights determination extreme learning machine algorithm, this study applies it to the classification and recognition of breast tumor diagnosis. Multiple distinct algorithms are employed to train and recognize the same breast tumor training and testing sets, which are then compared against the performance of the method proposed in this paper.

### 4.1 Experimental data

Data in this study were collected from an open data set published by the University of Wisconsin School of Medicine, including 569 cases of breast tumors, 357 benign and 212 malignant. In this paper, 450 groups of tumor data (282 benign cases, 168 malignant cases) were randomly selected as the training set, and the remaining 119 groups of tumor data (75 benign cases, 44 malignant cases) were selected as the test set. Each sample was composed of 30 data, including the mean, standard deviation and maximum value of 10 characteristic values extracted from the breast tumor sample data.

### 4.2 Experimental results and analysis

For the purpose of comparing algorithmic performance, three performance metrics were considered: the mean diagnostic rate for benign tumors (referred to as benign diagnosis rate), the mean diagnostic rate for malignant tumors (referred to as malignant diagnosis rate), and the average diagnostic accuracy rate. To ensure robustness of the comparison, independent experiments were conducted 20 times for each algorithm, including the proposed algorithm, V-ELM, Artificial Fish Swarm Algorithm-Extreme Learning Machine (AFSA-ELM), ELM, Learning Vector Quantization (LVQ), and Backpropagation Algorithm (BP). The average values of the benign diagnosis rate, malignant diagnosis rate, and overall accuracy rate were calculated and compared. It should be noted that the experimental results for V-ELM, AFSA-ELM, ELM, LVQ, and BP algorithms were sourced from Zhou and Yuan (2017). The comparative findings are summarized in Table 4.

**Table 4:** Performance comparison of multiple algorithms.

**Algorithm**	**Average classification accuracy (*%*)**	**Benign diagnosis rate (*%*)**	**Malignant diagnosis rate (*%*)**
V-DPELM	**98.32**	98.67	**97.73**
V-ELM	97.47	**99.93**	93.29
ELM	96.47	96.22	90.13
AFSA-ELM	96.59	96.38	90.61
LVQ	91.57	94.82	85.08
BP	85.88	84.87	88.93

Bold values indicate the maximum value.

From the findings presented in Table 4, it is apparent that the average accuracy rate achieved by the proposed algorithm surpasses that of the other algorithms. Although the benign diagnosis rate is slightly lower than that of the V-ELM algorithm, the malignant tumor diagnosis rate is considerably higher. These results highlight the efficacy of the proposed algorithm in rapidly and accurately identifying malignant tumors, thus mitigating the risks associated with delayed treatment and potential impacts on treatment efficacy resulting from misdiagnosis.

## 5 Conclusions

In the 12 randomly selected UCI datasets, the algorithm proposed in this paper, voting based double pseudo-inverse weights determination extreme learning machine algorithm, exhibits varying degrees of improvement in classification performance compared to the traditional V-ELM algorithm. Among these datasets, the Diabetes dataset shows the greatest increase in classification accuracy, with a significant enhancement of 10.27%. On the other hand, the LD dataset demonstrates the smallest improvement, with a marginal increase of only 0.09% in classification accuracy.

Moreover, the improved algorithm achieves optimal classification accuracy with fewer hidden layer neurons compared to the traditional ELM algorithm, resulting in a simpler network structure.

Additionally, the improved algorithm exhibits reduced variance and range in both the SL and Diabetes dataset experiments, indicating enhanced stability. Furthermore, in the breast tumor classification and recognition experiments, the diagnostic performance of the proposed algorithm surpasses that of V-ELM, AFSA-ELM, ELM, LVQ, and BP methods. This observation highlights the advantage of the proposed algorithm in achieving high classification accuracy in breast tumor auxiliary diagnosis. Thus, the application of this method for breast tumor auxiliary diagnosis is deemed feasible. In addition, it is worth pointing out that processing multi-dimensional data can be a research direction for future work.

## Data availability statement

Publicly available datasets were analyzed in this study. This data can be found here: https://archive.ics.uci.edu/datasets.

## Author contributions

RL: Funding acquisition, Investigation, Supervision, Validation, Writing—review & editing. LL: Conceptualization, Data curation, Formal analysis, Project administration, Resources, Software, Visualization, Writing—original draft, Writing—review & editing. BL: Investigation, Methodology, Writing—review & editing.
